# Worldwide Bronchiolitis obliterans research: A bibliometric analysis of the published literature between 2002 and 2022

**DOI:** 10.1097/MD.0000000000034263

**Published:** 2023-07-14

**Authors:** Zhengjiu Cui, Xu Zhou, Fei Luo, Jinjuan Wang, Juanjuan Diao, Yueli Pan

**Affiliations:** a First College of Clinical Medicine, Shandong University of Traditional Chinese Medicine, Jinan, China; b Department of Pediatrics, Affiliated Hospital of Shandong University of Traditional Chinese Medicine, Jinan, China.

**Keywords:** bibliometric analysis, bronchiolitis obliterans, collaboration, global research trends, research hotspots

## Abstract

Bronchiolitis obliterans (BO) is a rare and irreversible chronic respiratory disease. The diagnosis of BO is challenging, and there still needs to be specific therapies and uniform treatment guidelines available. Research on BO has grown steadily over the past 20 years, and with the continued interest of researchers in this area, a bibliometric study of BO becomes necessary. This topic aims to assess the current state of research in BO over the last 2 decades and to identify research hotspots and emerging directions. Information on BO-related articles were obtained from the Science Citation Index Expand of the Web of Science Core Collection (WOSCC [SCI-E]) database. Citespace (6.1.R6), VOSviewer (1.6.18), and the online bibliometrics website (https://bibliometric.com/) were used for bibliometric analysis mainly to include country/region, institution, author, journal, keywords, and references and to construct visual knowledge network diagrams. A total of 4153 publications from the WOSCC [SCI-E] database were included in this study. Most publications come from the United States, Japan, and Germany, which collaborate relatively more frequently. Research institutions in the United States, especially the University of Washington, published the largest number of BO-related articles. Regarding authors, Vos, R is the most productive author, while Verleden, GM is the most influential in BO. In addition, JOURNAL OF HEART AND LUNG TRANSPLANTATION is the journal with the most published articles. The most cited article is Estenne M, 2002. Based on the clustering analysis of keywords and references, the diagnosis of bronchiolitis obliterans syndrome (BOS), treatment of BOS, and risk factors of BO are the current research hotspots and future research trends. We analyzed the publication trends in BO by bibliometrics and mapped the knowledge network of major contributing countries/regions, institutions, authors, and journals. Current research hotspots were found based on the main keywords and references. The outcome may help researchers identify potential collaborators, collaborating institutions, and hot fronts in BO to enhance collaboration on critical issues and improve the diagnosis and treatment of BO.

## 1. Introduction

Bronchiolitis obliterans (BO) is a severe, irreversible chronic respiratory disease that is rarely seen clinically.^[1]^ It was first reported and standardized by German pathologist Lange in 1902.^[2]^ BO is mainly due to a variety of factors such as lung transplantation (LT), allogeneic bone marrow transplantation, infection, autoimmune diseases, inhalation of toxic gases, and adverse drug reactions leading to terminal and distal fine bronchial injury, inflammation and fibrosis, resulting in slight airway narrowing or complete obstruction.^[3,4]^ Patients present to have symptoms of persistent or recurrent cough (more than 6 weeks), wheezing, shortness of breath, dyspnea, exercise intolerance, etc.^[5]^ BO often affects the pediatric population and is most commonly associated with post-infection bronchiolitis obliterans (PIBO), where the majority of pathogens are viral, with adenovirus infection predominating.^[6]^ Adult onset is more associated with occupational inhalation injury, allergic pneumonia, and autoimmune disease.^[7]^ Clinicians usually diagnose BO based on a combination of typical symptoms, pulmonary function, and radiological findings, but lung biopsy and histopathology are the gold standards for diagnosis.^[8]^ General supportive treatment for BO is common and vital in clinical practice. Examples of such treatment include avoiding inhalation of irritants, getting influenza vaccination on time, implementing effective exercise and fitness, keeping the airway open, and maintaining adequate nutrition and oxygen in the body.^[7]^ The prognosis of BO is uncertain, and the disease is prone to recurrence or exacerbation, which brings significant economic and psychological stress to patients and families. Therefore, it is necessary to analyze the global BO research trends and frontier hotspots using bibliometrics.

In recent years, bibliometrics has been widely used in many fields, including medicine.^[9–11]^ It excels in exploring the underlying knowledge structures in academic literature and integrating visualization results to analyze important information in the field further. BO research has been extended to many areas of medicine, including the respiratory,^[12]^ digestive,^[13]^ circulatory,^[14]^ and urinary^[15]^ systems. Bibliometrics is a vast knowledge integrating mathematics, statistics, and literature.^[16,17]^ This quantitative analysis strategy provides an objective and comprehensive overview to discover the spatial and temporal distribution of the research field, reveals hot and emerging topics based on multiple indicators such as references, keywords, authors, journals, countries, and institutions, and ultimately contributes to the continuous advancement of the research field.^[18–20]^ It has been noted that tracking knowledge diffusion and cluster analysis can lead to more comprehensive results in interdisciplinary studies.^[21,22]^ Only some existing BO-related meta-analyses have resulted in widely varying outcome indicators and unclear conclusions due to the insufficient number and low quality of included publications. In contrast, most reviews use a meta-based approach that can only elucidate the progress of BO in specific and limited aspects at the level of evidence-based medicine and cannot provide an overview of all studies in the field of BO. Bibliometric analysis can provide visual graphics that reveal the current status and evolution of research topics from a more systematic, visual, and comprehensive perspective.^[23]^

Over the past 20 years, a great deal of research has been conducted on BO by researchers and clinicians. Although some literature reviews and meta-analyses have presented comprehensive and concise conclusions, the continuously growing number of publications makes it difficult for scholars to identify research hotspots and frontiers from the growing body of literature. To our knowledge, there is no bibliometric analysis of BO research. Therefore, this study provides statistical analysis and visualization of the global literature on BO based on the Web of Science Core Collection [Science Citation Index Expanded] (WOSCC [SCI-E]) for the period 2002 to 2022. Our objectives are to provide an overview of the trends in the field of BO in the past 20 years; summarize the popular research clusters and directions in the field of BO; look forward to the future development of the field of BO.

## 2. Materials and methods

### 2.1. Data sources

**Figure 1.**
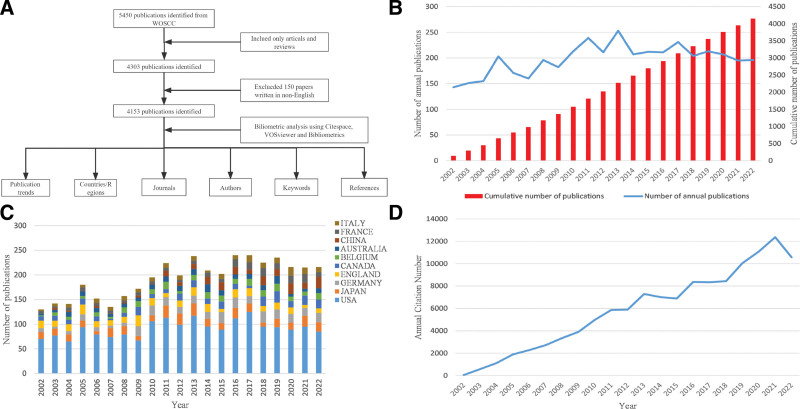
Research methodology and publication trend charts. (A) Flowchart of the study. (B) Overall trends in global publications from 2002 to 2022. (C) Trends in annual publications in the top 10 countries/regions. (D) Annual citation trends.

The literature we included in the study was obtained from WOSCC [SCI-E]. We use the database WOSCC [SCI-E] because it covers authoritative, high-impact scholarly journals worldwide, contains comprehensive citation indexing records, and focuses on the natural sciences.^[24]^ Some studies have shown that WOS has higher accuracy and period than Scopus and other databases.^[25]^ Although PubMed is the central biomedical search platform, it is a typical abstract-based database and lacks citation information.^[26]^ WOSCC [SCI-E] is considered by scholars to be the most appropriate database for bibliometric analysis.^[27]^ All data in this study were retrieved and downloaded on January 15, 2023, with the following search strategy: TS = (obliterative bronchiolitis AND BO AND constrictive bronchiolitis AND popcorn lung). The time limit was from January 1, 2002 to December 31, 2022. Only research and review articles published in English were selected. The detailed flow of the search is shown in Figure 1A. Two researchers (C and W) independently analyzed the data, including titles, abstracts, keywords, authors, institutions, countries/regions, journals, references, and citations of publications, and if there were differences of opinion, they were resolved by independent consultation and discussion by a third researcher (Diao). A total of 4153 documents were included in the study, and the “full record and cited references” of the documents were derived in “plain text” form.

### 2.2. Data analysis

We exported global annual publication counts, cumulative publication counts, annual citation counts, and annual publication counts by country/region from the WOSCC [SCI-E] database, aggregated the statistics using Microsoft Excel 2019, and created graphs. The “plain text” data were imported into Citespace (6.1.R6) and VOSviewer (1.6.18). Countries were aggregated by combining Taiwan, Hong Kong, Macao, and Peoples R China into CHINA, Scotland, England, Wales, and Northern Ireland into the United Kingdom. Citespace is a Java-based bibliometric analysis visualization software developed by Dr Chaomei Chen.^[28]^ The time span (January 2002–December 2022), years per slice (1), links (strength: cosine, scope: within slices), selection criteria (maximum number of selected items per slice = 50), pruning (Pathfinder, Pruning sliced networks), and all of the other parameters were left at their default settings. The Node Type parameter area is set as follows: Select “Institution,” “Keyword,” and “Reference” for co-occurrence analysis and burst detection. VOSviewer is a sociometric network analysis software developed by Leiden University.^[29]^ In this study, the parameters of the VOSviewer were as follows: The counting method was selected for “full counting.” The minimum number of citations for the co-cited authors and co-cited references was 20 and 30, respectively. The unit of analysis of the co-occurrence keyword was “all keyword,” and the threshold for the minimum number of occurrences was set to 100. The thresholds for the minimum number of occurrences of co-occurring institutions and co-occurring authors were set to 15 and 15. We also used the online bibliometrics website (https://bibliometric.com/) to visualize international collaboration between countries and to count the number of publications from major countries. The platform uses the functions of the “Partnership Collaboration” and “Total Literature Collaboration” modules for mapping and data extraction.

## 3. Results

### 3.1. Global publication trends

**Figure 2.**
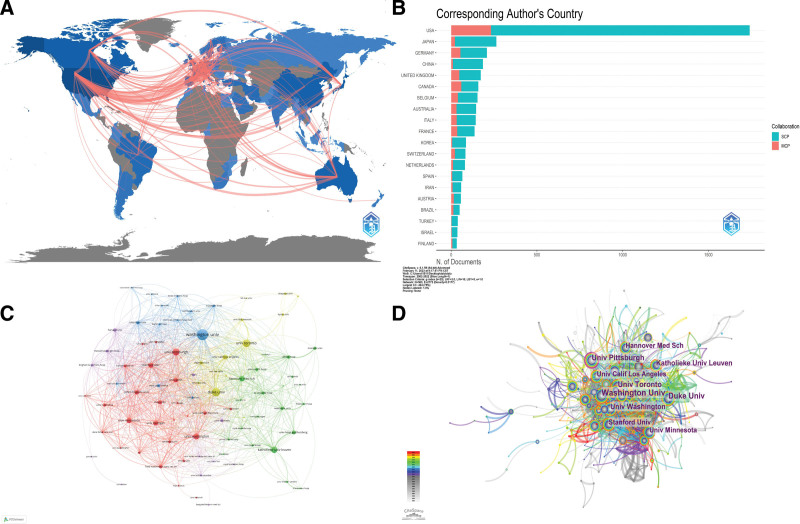
Visualization of countries/regions and institutions. (A) World Collaborative Relationships Map. (B) Comparison of single-country publications and multiple country publications from different countries. MCP = multiple country publications, SCP = single-country publications. (C) VOSviewer network map of institutions involved in BO. (D) CiteSpace network map of institutions involved in BO. BO = Bronchiolitis obliterans.

As shown in Figure 1B and D, the total number of publications and citations of BO have increased significantly in the last 20 years. The annual number of publications has changed in 3 phases: a period of rapid growth (2002–2005), a period of fluctuating increase (2006–2013), and a period of stable equilibrium (2014–2022). The annual number of publications exceeded 200 for the first time in 2010. It peaked (253 articles) in 2013 and has remained around 200 articles since then. Figure 1C depicts the trend of the total number of publications in the top 10 countries/regions between 2002 and 2022, which is consistent with the annual trend of global publications in Figure 2B, indicating the dominance of these countries/regions in the field of BO research, with the US leading the way.

### 3.2. Distribution of countries and institutions

According to statistics, 72 countries/regions and 560 institutions worldwide have participated in BO studies in the last 20 years. Figure 2A shows a strong collaboration among countries/regions such as USA, UK, JAPAN, and AUSTRIA. As shown in Figure 2B, the USA ranks first in the number of single-country and multi-country publications among different countries/regions. The top 10 countries/regions with the highest number of publications and highest centrality are listed in Table 1. The country/region with the highest number of publications is the USA (n = 1914), followed by JAPAN (n = 337), and GERMANY (n = 305). The centrality index measures the prominence of the network nodes, with the USA (0.46) being the core of the network, followed by the UK (0.23) and SPAIN (0.12). The top 10 institutions in terms of number of publications and centrality ranking are listed in Table 2 and visualized in Figure 2C and D. The University of Washington (n = 212) published the most papers, followed by Duke University (n = 155) and the University of Toronto (n = 140). The University of Pittsburgh has the highest centrality (0.1), visible as a purple circle in Figure 2D, indicating some influence in the field. All other institutions have low centrality, and the analysis of inter-institutional co-authorship yields 5 clusters (Fig. 2C), where the size of the circles indicates the number of publications and the thickness of the line indicates the strength of the connection, and the top 5 institutions in terms of several publications are separated into 4 larger clusters. However, it shows that there needs to be more collaboration between them.

**Table 1:** The top 5 countries/regions with the highest number of publications and highest centrality.

Rank	Countries	Publications	Countries	Centrality
1	USA	1914	USA	0.46
2	JAPAN	337	UK	0.23
3	GERMANY	305	SPAIN	0.12
4	UK	276	ITALY	0.09
5	CANADA	255	AUSTRIA	0.09

**Table 2:** The top 5 institutions with the highest number of publications and the highest centrality.

Rank	Institutions	Country	Publications	Institutions	Country	Centrality
1	University of Washington	USA	212	University of Pittsburgh	USA	0.1
2	Duke University	USA	155	University of Toronto	CANADA	0.08
3	University of Toronto	CANADA	140	Hannover Medical School	GERMANY	0.08
4	University of Pittsburgh	USA	118	Stanford University	USA	0.08
5	Katholieke Universiteit Leuven	BELGIUM	108	University of Washington	USA	0.07

### 3.3. Distribution of authors and co-cited authors

**Figure 3.**
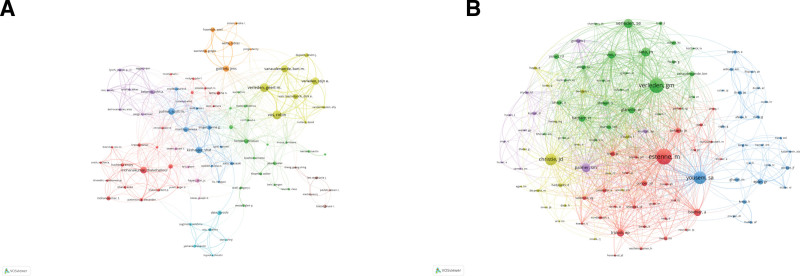
Author visualization. (A) Visualization of authors. (B) Visualization of co-cited authors.

Table 3 lists the top 5 most published and cited co-citation authors. These authors are mainly from BELGIUM and USA. However, BELGIUM is only ranked 6th in the number of publications and has low centrality. Vos, R is the most prolific author with 77 publications, followed by Verleden, GM (74 publications), and Verleden, S (61 publications). The collaborative network between authors is shown in Figure 3A, where the top 4 authors, in terms of the number of publications, are in the yellow cluster, with thicker connecting lines between them, indicating close collaboration. There are geographical differences between clusters, and the linkage needs to be improved. Co-citation analysis refers to 2 authors or papers having a co-citation relationship when they are cited by a third author or paper simultaneously. The degree of citation is a vital indicator of an author contribution. The results of the co-citation analysis visualization (Fig. 3B) showed that Estenne, M (1104 times), Verleden, GM (1044 times), and Yousem, SA (882 times) had the most co-citations and their clusters accounted for the majority of them. The above results indicate that these authors are very interested in BO-related research.

**Table 3:** Top 5 authors and co-cited authors.

Rank	Authors	Counts	Citations	Country	Co-cited authors	Co-citations	Country
1	Vos, R	77	2785	BELGIUM	Estenne, M	1104	BELGIUM
2	Verleden, GM	74	3621	BELGIUM	Verleden, GM	1044	BELGIUM
3	Verleden, S	61	1998	BELGIUM	Yousem, SA	882	USA
4	Vanaudenaerde, BM	58	2072	BELGIUM	Christie, JD	810	USA
5	Mohanakumar, T	54	1853	USA	Verleden, S	637	BELGIUM

### 3.4. Distribution of journals and co-cited journals

**Figure 4.**
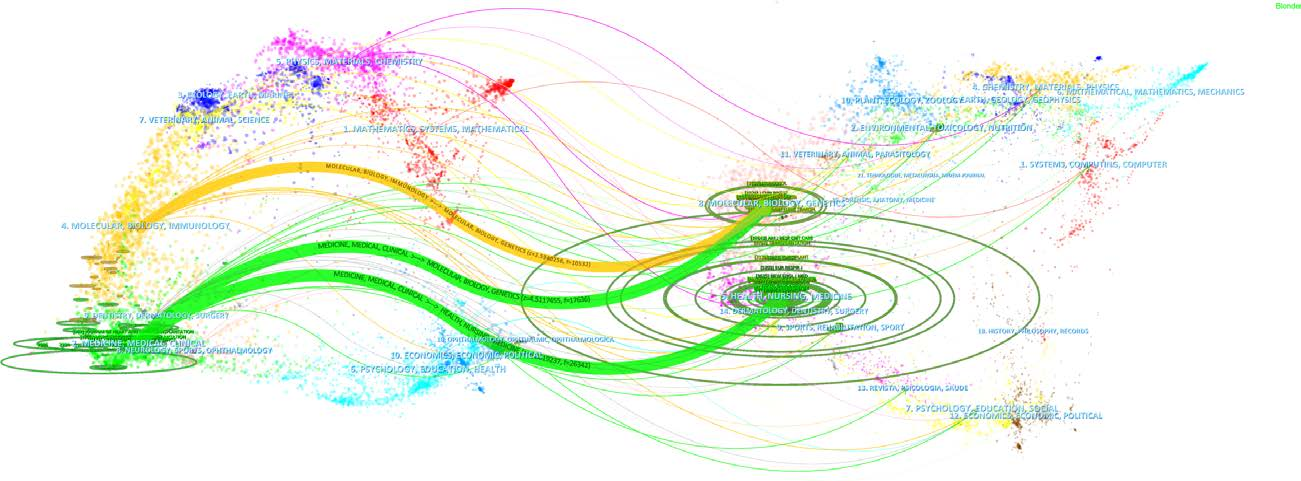
The dual-map overlay of BO research. BO = Bronchiolitis obliterans.

We performed a visual analysis of the journals, and the top 5 results are summarized in Tables 4 and 5. JOURNAL OF HEART AND LUNG TRANSPLANTATION had the highest number of papers (n = 352, 8.47%), followed by the AMERICAN JOURNAL OF TRANSPLANTATION (n = 199, 4.79%) and TRANSPLANTATION (n = 198, 4.76%). AMERICAN JOURNAL OF RESPIRATORY AND CRITICAL CARE MEDICINE had the highest co-citation frequency (n = 2898), and the remaining 4 journals had more than 2000 co-citations. According to the Journal Citation Report 2021, 90% of the journals are in the Q1 region. JOURNAL OF HEART AND LUNG TRANSPLANTATION, TRANSPLANTATION and AMERICAN JOURNAL OF RESPIRATORY AND CRITICAL CARE MEDICINE is the leading journal in terms of the number of articles issued and co-citation frequency, contributing to the field of BO. The double graph overlay of journals shows citing journals on the left and cited journals on the right side, and the colored paths between them indicate citation relationships. The green path in Figure 4 indicates that literature in molecular/biology/genetics/nursing/health/medical journals is frequently cited in medical/clinical journals.

**Table 4:** The top 5 journals in terms of number of publications.

Rank	Journal	Publications	IF (2021)	JCR
1	JOURNAL OF HEART AND LUNG TRANSPLANTATION	352 (8.47%)	13.569	Q1
2	AMERICAN JOURNAL OF TRANSPLANTATION	199 (4.79%)	9.369	Q1
3	TRANSPLANTATION	198 (4.76%)	5.385	Q1
4	TRANSPLANTATION PROCEEDINGS	114 (2.74%)	1.014	Q4
5	AMERICAN JOURNAL OF RESPIRATORY AND CRITICAL CARE MEDICINE	95 (2.28%)	30.528	Q1

**Table 5:** Top 5 co-cited journals in terms of total citation frequency.

Rank	Co-cited journal	Citation	IF (2021)	JCR
1	AMERICAN JOURNAL OF RESPIRATORY AND CRITICAL CARE MEDICINE	2898	30.528	Q1
2	JOURNAL OF HEART AND LUNG TRANSPLANTATION	2609	13.569	Q1
3	CHEST	2399	11.393	Q1
4	TRANSPLANTATION	2261	5.385	Q1
5	EUROPEAN RESPIRATORY JOURNAL	2031	33.801	Q1

### 3.5. Analysis of keywords

**Figure 5.**
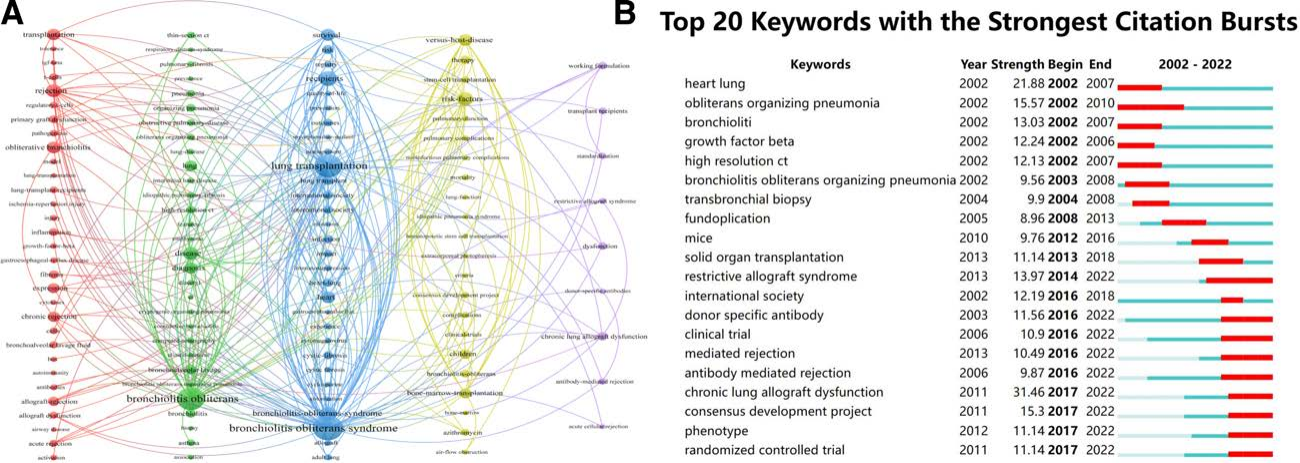
Keywords visualization. (A) Visualization of keywords. (B) The top 20 keywords with the strongest citation bursts.

By analyzing the keywords included in the literature, we see the top 20 keywords with the highest frequency of occurrence in Table 6, with bronchiolitis obliterans syndrome (BOS) (n = 1587), BO (n = 1167), LT (n = 1128), recipient (n = 476), and risk factor (n = 452) ranked in the top 5, and their centrality was more significant than 0.1. By limiting the number of keyword occurrences to more than 55, we obtained 122 keywords and used VOSviewer for cluster analysis and visualization, as shown in Figure 5A, and finally obtained 5 clusters. Cluster 1 (red) mainly includes the pathogenesis of BO, such as ischemia-reperfusion injury, inflammation, fibrosis. Cluster 2 (green) mainly deals with diagnosing and differentiating BO, such as high-resolution ct, clinical-features, respiratory-distress-syndrome. Group 3 (blue) is mainly related to the effect of BO on the organism and prognosis, such as heart-lung, gastroesophageal-reflux, adult lung. Group 4 (yellow) is mainly related to the risk factors and treatment of BO, such as versus-host-disease, stem-cell transplantation, clinical-trials. Group 5 (purple) mainly includes transplants related to BO, such as chronic lung allograft dysfunction (CLAD). As shown in Figure 5B, 20 keywords have the most prolonged duration among all keywords. From 2002 to 2018, research hotspots mainly focused on exploring BO-related diseases involving cardiopulmonary and gastrointestinal diseases. From 2018 to 2022, the research hotspots in BO shifted to transplantation and clinical trials. The 3 prominent terms with the highest intensity were CLAD (31.46), heart-lung (21.88), and obliterans organizing pneumonia (15.57).

**Table 6:** The top 20 most frequently occurring keywords.

Rank	Keyword	Occurrences	Centrality	Rank	Keyword	Occurrences	Centrality
1	Bronchiolitis obliterans syndrome	1587	1.52	11	Bone marrow transplantation	269	0.18
2	Bronchiolitis obliteran	1167	0.29	12	Rejection	242	0.48
3	Lung transplantation	1128	0.45	13	Diagnosis	236	0.38
4	Recipient	476	0.17	14	Survival	227	0
5	Risk factor	452	1.22	15	Chronic rejection	207	0.54
6	Disease	372	0.38	16	Expression	176	0.12
7	International society	300	0.54	17	Allograft rejection	164	0.29
8	Obliterative bronchioliti	284	0.12	18	Cystic fibrosis	163	0
9	Vs host disease	276	0	19	Lung transplant	144	0
10	Heart	269	0.29	20	Chronic lung allograft dysfunction	139	0.06

### 3.6. Analysis of references

**Figure 6.**
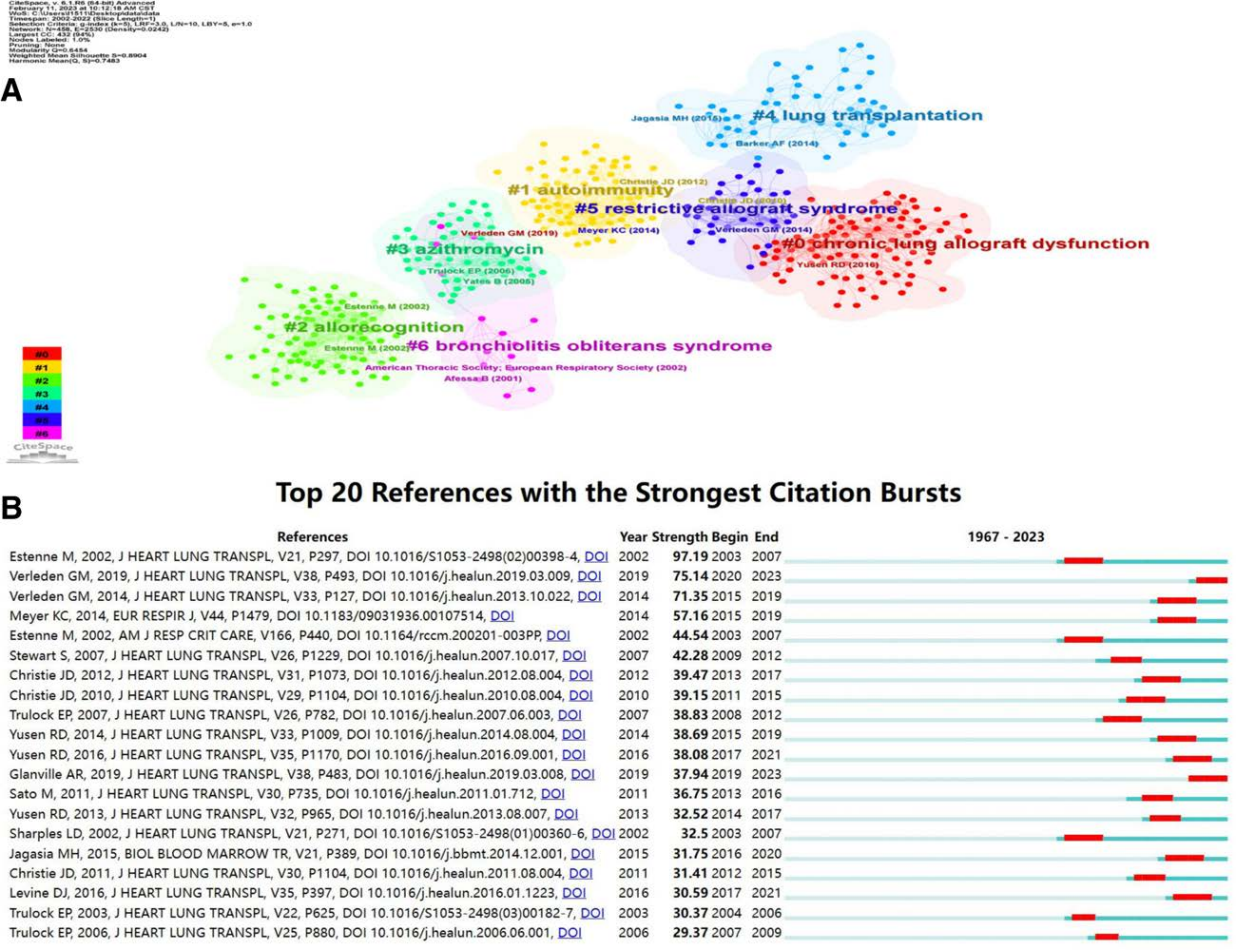
References visualization. (A) Reference co-citation network for clustering of title terms. (B) The top 20 cited references with the strongest citation bursts.

The top 10 most cited articles in the BO research area are shown in Supplementary Table 1, http://links.lww.com/MD/J265, with the top article cited 201 times, titled “Bronchiolitis Obliterans Syndrome 2001: An Update of the Diagnostic Criteria." The International Society for Heart and Lung Transplantation has constructed a uniform system based on the measurement of forced expiratory volume in 1 second (FEV1) and introduced the term BOS in recipients with persistently declining allograft function after LT, which is challenging to confirm accurately in bronchopulmonary biopsy specimens.^[30]^ The contribution of this article was to update the diagnosis of BOS. Sometime later, researchers found that many allograft or extra-graft abnormalities may also cause a persistent decline in FEV1, suggesting that BOS represents more than irreversible airway obstruction. The second-ranked article proposed a definition of CLAD that may help identify different phenotypes in lung transplant recipients, titled “A new classification system for chronic lung allograft dysfunction.”^[31]^ CiteSpace with setting node type = cited reference was used for co-cited reference clustering analysis, and other parameters were set to default values. We obtained a total of 7 clusters that demonstrate the research themes in BO (Fig. 6A), mainly focusing on allograft recognition and autoimmunity. Figure 6B shows the top 20 references with the strongest citation bursts, with the 2 articles published in 2019 bursting with intensity to the present.

## 4. Discussion

The bibliometric analysis of global BO paper data provides a very intuitive picture of the trends in the field, and these results can provide scientific guidance for researchers to conduct more in-depth studies in BO and help clinicians to have a better understanding of BO. Although BO is a rare disease, the number of publications per year has generally increased between 2006 and 2013, with 4153 relevant publications in the past 20 years, indicating a productive research output in BO. The number of relevant articles has remained roughly stable in recent years. However, it has decreased from the previous years, especially after 2019, which we speculate is due to the increased awareness of protection and reduced incidence of BO after infection following the global epidemic of novel coronaviruses. The rapid increase in citations reflects the increasing number of researchers interested in BO.

In terms of countries/regions, the USA ranks first in terms of the number of publications and centrality, indicating that it has made significant contributions to BO research and frequently cooperates with other countries. JAPAN and GERMANY have more articles but have very low centrality and lack cooperative exchanges. USA, CANADA, GERMANY, and UNITED KINGDOM have a high number of publications by several countries in collaboration and are potential BO research centers and research highlands. Three of the top 5 institutions with the highest number of publications are from the USA, and 4 of the top 5 lead authors in BO are from BELGIUM. Authors and institutions from the USA and European countries dominate BO research. Most of the major research institutions in BO are concentrated in universities, and they have a low centrality, and cooperation needs to be strengthened. Hospitals, as prominent institutions of clinical research, need to make more breakthroughs and achievements in BO research in the future. Although Vos, R is the most prolific author, he serves only as a marginal contributor. verleden, GM has 74 publications (ranked second), 3621 total citations (ranked first), and 1044 co-citation numbers (ranked second); in contrast, he is more influential in this research area.

Four of the top 5 journals in terms of the number of publications have JCR partitions in Q1, and the top 5 cited journals in total citations have JCR partitions in Q1, indicating that these core journals in the field of BO have a great influence. The JOURNAL OF HEART AND LUNG TRANSPLANTATION has 352 articles (ranked first) and 2898 citations (ranked second). 8 of the top 10 highly cited articles are from the JOURNAL OF HEART AND LUNG TRANSPLANTATION, which proves that it is the most authoritative journal in the field of BO research. In addition, TRANSPLANTATION and AMERICAN JOURNAL OF RESPIRATORY AND CRITICAL CARE MEDICINE are among the top 5 journals in terms of number of articles and citations, and they are also one of the influential journals in this field.

## 5. Hotspots and frontiers

Based on an effective combination of popular keywords, keyword bursts, keyword clustering and reference analysis, we found that the rarity of BO has left its pathogenesis under-studied.^[32]^ However, the emergence of LT and hematopoietic stem cell transplantation (HSCT) as therapeutic modalities has increased scientists’ interest in this rare disease and has provided many research results as a result.^[33]^ Current hot spots and future research trends in this field focus on the diagnosis and treatment of transplant-related BO. Clinically, the disease with clinical manifestations, imaging, and pulmonary function tests consistent mainly with BO after transplantation is uniformly named BOS. The diagnosis of BOS, treatment of BOS, and risk factors for BO are summarized below.

1. Diagnosis of BOS. BOS is the most common form of CLAD characterized by airflow limitation and obstructive spirometry patterns. The airflow limitation is measured by a ratio of FEV1 to forced vital capacity <0.7, where a ≥20% decrease in FEV1 in spirometry is an important part of the definition.^[34]^ Computed tomography and micro-CT analysis show many small airway obstructions below the fifth-generation airway branches, with 40% to 70% of the airway affected to varying degrees, an outcome not explained by acute rejection, infection, or other coexisting disease.^[35]^ Combining quantitative parametric response mapping with qualitative CT image features can improve diagnostic level for BOS.^[36]^ One study found damage to the pIgR/IgA system in patients with BOS, and it was hypothesized that patient morbidity was associated with an increased likelihood of local infection.^[37]^ Plasma levels of CRH, FERC2, IL-20RA, TNFB, and IGSF3 are significantly lower in patients with BOS, whereas MMP-9, CTSL1, and IL-26 are elevated, and these indicators are expected to provide a basis for the diagnosis and identification of BOS.^[38,39]^ Histopathologically, patients with BOS patients have different characteristics than non-CLAD patients in terms of exoskeletal lung tissue: patients with confirmed BOS have significant histological lesions, mainly centered in the airways; significant vascular lesions in BOS; fibrotic changes in BOS; and non-CLAD patients exhibit airway-centered lesions with lesser degrees of vascular lesions and fibrotic changes.^[36]^ BOS after HSCT triggers systemic graft-versus-host disease (GVHD), and the effects of BOS after LT are limited to lung allografts, the former being more challenging to diagnose. Studies on blood genes suggest that POU2AF1, TCL1A, and BLK can be used as predictive biomarkers for BOS, which promises to bring forward preventive treatment measures to minimize morbidity.^[40]^
2. Treatment of BOS. Firstly, timely treatment of BOS triggers or underlying diseases is required. The second is to optimize the patient immunosuppressive regimen. Immunosuppression is the primary therapeutic drug choice for BOS. Systemic corticosteroids have become an essential support for BOS treatment but are not easily used for a long time because of their side effects, and others include tacrolimus, cyclosporine, and mycophenolate.^[41,42]^ Ruxolitinib is an effective anti-GVHD agent, and pirfenidone has anti-inflammatory and anti-fibrotic effects, with improved pulmonary function tests and reported outcomes in patients after application supporting its addition to treating BOS. However, there is a lack of prospective randomized controlled trial validation.^[43]^ In patients with severe symptoms of dyspnea, inhaled corticosteroids combined with long-acting bronchodilators are added. More evidence supports that short- to medium-term application of azithromycin effectively improves lung function and results in a higher survival benefit in patients with BOS after LT.^[33,44–46]^ The leukotriene antagonist montelukast has also been used in the clinic as it has shown antifibrotic effects in animal models of BOS.^[47]^ Calcineurin inhibitors, Rituximab, Etanercept, and Tyrosine kinase inhibitors are also used clinically for BOS because they inhibit fibrosis via the platelet-derived growth factor pathway.^[48]^ In recent years, second-line treatments for BOS have included extracorporeal photopheresis or TLI cell therapy, which can induce immune tolerance and inhibit the progression of pulmonary fibrosis. If the disease progresses or worsens despite treatment, retransplantation remains the ultimate necessary option for BOS.^[49]^
3. Risk factors for BO. Hypoxemia, mechanical ventilation, shortness of breath, and wheezing are the main risk factors for PIBO. In contrast, glucocorticoids, gamma globulin, bacterial co-infection, and a history of wheezing and male are also considered to be among the contributing factors.^[50]^ Lactatedehydrogenase levels, pleural effusion, hypoxemia, gender, and mechanical ventilation have been noted as risk factors for PIBO in Korean patients.^[51]^ Persistent wheezing and acute respiratory failure in Chinese children with adenovirus pneumonia is an independent risk factor for PIBO.^[52]^ Risk factors for developing BO after LT include alloimmune-induced autoimmunity, acute rejection, viral infections, bacterial and fungal infections, primary graft dysfunction, and gastroesophageal reflux disease.^[34]^ The development of BO after HSCT is associated with higher pre-transplant cyclophosphamide exposure, no anti-thymocyte globulin pretreatment regimen, moderate to severe chronic GVHD, and peripheral blood as a stem cell source.^[53,54]^ Risk factors may vary by age, region, and ethnicity.^[55]^ Recent literature on risk factors contains small sample sizes, covers limited regions, and has not been analyzed for ethnic differences, reflecting the inability to share patient data between countries and institutions and the imperfect mechanism of cooperative communication. We believe that countries and institutions with high volume and quality of publications should take the lead in establishing a global BO cooperative alliance, organize regular online and offline communication and training, establish a BO resource database, and form a multicenter study scale to make the risk factors analyzed more representative and universal.

## 6. Advantages and limitations

No researcher has published a bibliometric analysis of BO research. This study uses Citespace (6.1.R6) and VOSviewer (1.6.18) to summarize comprehensive information and hot frontiers of BO from a bibliometric analysis perspective, providing a meaningful reference for research in this field. This study is not quite perfect and has limitations. First, we did not collect literature from other databases such as Pubmed, Embase, and Scopus. Secondly, we only kept the literature whose language was English. Finally, VOSviewer and CiteSpace cannot perform advanced statistical analysis, which may introduce statistical bias. However, these limitations do not affect the comprehensive information extraction and analysis of the BO field.

## 7. Conclusion

We summarized and analyzed the literature on BO worldwide between 2002 and 2022. The results show that much high-quality literature on BO have been published in the past 20 years, and research depth and breadth have expanded. Using the visualization functions of VOSviewer and CiteSpace, the countries/regions, institutions, journals, and authors that have contributed to this field were identified, and the cooperation among them is beneficial to the progress and development of the discipline. We believe these outstanding contributors should form an international exchange organization for BO to realize the dissemination and research results through mutual personnel visits, resource sharing, and research assistance. We found that the diagnosis of BOS, treatment of BOS, and risk factors of BO are hot and cutting-edge topics in this field. Researchers should pay more attention to the relationship between transplantation and BO and transform treatment into prevention by studying risk factors for BO in the global population.

## Author contributions

**Conceptualization:** Zhengjiu Cui.

**Data curation:** Xu Zhou, Fei Luo.

**Supervision:** Jinjuan Wang.

**Writing – original draft:** Zhengjiu Cui.

**Writing – review & editing:** Juanjuan Diao, Yueli Pan.

## Supplementary Material


